# Pyrethroid and DDT Resistance and Organophosphate Susceptibility among *Anopheles* spp. Mosquitoes, Western Kenya

**DOI:** 10.3201/eid2112.150814

**Published:** 2015-12

**Authors:** Christine L. Wanjala, Jernard P. Mbugi, Edna Ototo, Maxwell Gesuge, Yaw A. Afrane, Harrysone E. Atieli, Guofa Zhou, Andrew K. Githeko, Guiyun Yan

**Affiliations:** Kenyatta University, Nairobi, Kenya (C.L. Wanjala, J.P. Mbugi, E. Ototo);; Kenya Medical Research Institute, Kisumu, Kenya (C.L. Wanjala, E. Ototo, M. Gesuge, Y.A. Afrane, H.E. Atieli, A.K. Githeko);; Masinde Muliro University of Science and Technology, Kakamega, Kenya (C.L. Wanjala);; Program in Public Health, University of California, Irvine, USA (G. Zhou, G. Yan)

**Keywords:** *Anopheles*, *gambiae*, sensu lato, sensu stricto, *arabiensis*, mosquitoes, malaria, vector-borne infections, insecticide resistance, susceptibility, pyrethroid, DDT, organophosphate, carbamate, Kenya, Africa

## Abstract

We conducted standard insecticide susceptibility testing across western Kenya and found that the *Anopheles gambiae* mosquito has acquired high resistance to pyrethroids and DDT, patchy resistance to carbamates, but no resistance to organophosphates. Use of non–pyrethroid-based vector control tools may be preferable for malaria prevention in this region.

During the past decade, a massive scale-up of insecticide-treated nets (ITNs) and indoor residual spraying (IRS) of insecticides in malaria-endemic areas worldwide have led to a substantial reduction in mosquitoes and, paired with the use of artemisinin combination treatments, in overall malaria prevalence and incidence (*1*). However, although most studied sites showed sustained low-level transmission, other sites had stable or resurging malaria cases and vector populations (*2*–*5*). It is generally believed that the recent resurgence in malaria was caused in part by increased vector resistance to pyrethroid insecticides related to the intensive use of ITNs and IRS (*6*–*8*). Insecticide resistance is among the most critical challenges in malaria control. Although several new insecticides have been tested as alternatives to pyrethroids for IRS, there is strong debate among decision makers at the national level on whether to implement IRS and which insecticides should be used. Comprehensive evaluation of insecticide resistance across different malaria-endemic areas will provide critically needed data on use of new IRS strategies as alternative malaria control tools for further reducing malaria incidence in Africa.

## The Study

**Figure 1:**
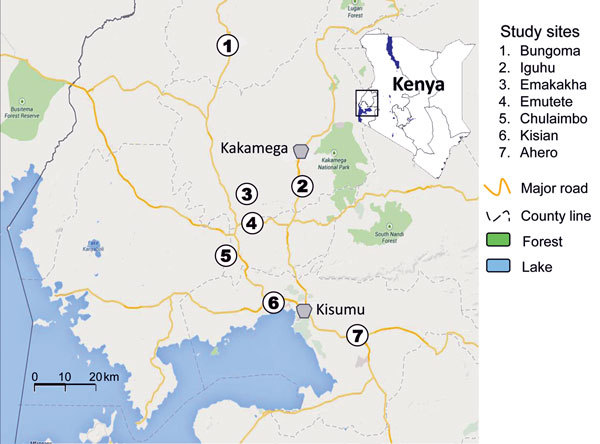
Study sites (circles) for discerning the presence of pyrethroid and DDT resistance and organophosphate susceptibility among *Anopheles* spp. mosquitoes, western Kenya, 2012–2013.

During April 2012–July 2013, we conducted this study in 7 sentinel sites across different malaria-endemic zones in western Kenya (Figure 1). Malaria vector dynamics and parasite prevalence have been studied in 3 sites (*9*), and ITN coverage was generally >80% (*10*). Bungoma, Emutete, Iguhu, and Emakakha are in the highland-fringe malaria epidemic area; Chulaimbo, Ahero, and Kisian are in the malaria-endemic basin region of Lake Victoria (lowland). All sample sites were in rural or suburban areas. 

Agricultural and public health use of insecticides in each study site was surveyed by using questionnaire surveys in 30 randomly selected households per site. Mosquito larvae were collected from each study site, fed with TetraMin fish food (Spectrum Brands, Inc., Blacksburg, VA, USA), and raised to adults in the insectary at the Kenya Medical Research Institute in Kisumu. The insectary was not regulated for temperature and humidity; ambient temperature (average ≈24°C) and humidity (≈75% relative humidity) were used for the study. Emerged adults were fed with 10% sucrose solution, and 2- to 5-day-old females were used to determine insecticide susceptibility by using the standard World Health Organization (WHO) insecticide susceptibility tube test http://www.who.int/malaria/publications/atoz/9789241505154/en/. Four classes of insecticides were tested, including pyrethroids lambdacyhalothrin (diagnostic dose 0.05%), deltamethrin (4%), and permethrin (0.75%); organochlorine DDT (0.05%); organophosphate malathion (5%); and carbamate bendiocarb (0.1%) (*11*). The WHO-designated, pyrethroid-susceptible *An. gambiae* mosquito in Kisumu was used as a control. 

Mosquitoes were exposed to each insecticide for 1 h and then maintained in holding tubes with 10% sucrose solution for 24 h. Mortality rates were scored after the 24-h recovery period; the susceptibility status of the mosquito populations was graded according to WHO criteria (*11*). Knockdown time (time required to render an adult mosquito unable to fly) was recorded every 10 minutes. Tests were done at 26°C ± 2°C and 80% ± 10% relative humidity during the 1-h exposure period and the subsequent 24-h period during which the mosquito would die or recover, with a 12D:12N photoperiod. We tested 200 mosquitoes per site per insecticide; that is, 8 replicates of exposure and 2 replicates of control, with 20 mosquitoes per replicate. A total of 8,400 (200 per site × 7 sites × 6 insecticides) female mosquitoes were tested. Knockdown rates are provided in the Technical Appendix.

We identified species of a subset of randomly selected susceptible and resistant mosquitoes from the bioassay by using 16s rDNA PCR (*12*). A total of 1,002 specimens were molecularly identified. The real-time TaqMan assay was used to detect knockdown resistance (*kdr*) gene mutations and genotypes at amino acid position L1014 of the voltage-gated sodium channel gene (*12*). A total of 579 mosquitoes were examined for *kdr* mutation.

**Figure 2:**
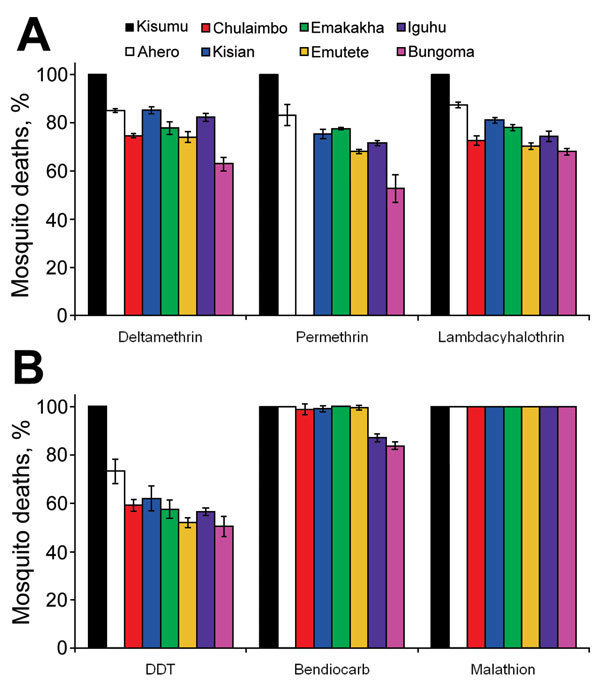
*Anopheles gambiae *sensu lato mortality rates associated with various insecticides and study sites, western Kenya. A) Mortality rates associated with pyrethroid insecticides deltamethrin, permethrin, and lambdacyhalothrin. In Chulaimbo, permethrin was not tested because of a lack of mosquitoes. B) Mortality rates associated with DDT (organochlorine), bendiocarb (carbamate), and malathion (organophosphate). The susceptible Kisumu strain at Kenya Medical Research Institute was used as a control. Error bars indicate 95% CIs.

Results of WHO susceptibility bioassays showed a 100% mortality rate in the susceptible Kisumu *An. gambiae* reference strain after exposure to all insecticides tested and 50.4%–87.2% in the 7-field *An. gambiae* sensu lato populations resulting from exposure to all pyrethroids and DDT. These field populations were highly resistant to pyrethroids, demonstrated by an observed mortality rate that was considerably less than the WHO 90% threshold for resistance. The Bungoma population was the most resistant, exhibiting only a 50% mortality rate against permethrin. The WHO susceptibility bioassay also indicated that *An. gambiae* sensu lato was highly resistant to DDT: mortality rates ranged 50.4%–73.2% at all sites (Figure 2). Five of the 7 study populations were susceptible to bendiocarb, but 2 populations (Iguhu and Bungoma) were resistant (Figure 2). A 100% mortality rate was observed in mosquito populations exposed to malathion at all sites (Figure 2).

PCR analysis found that *An. gambiae* sensu stricto (s.s.) was the predominant species in Chulaimbo (72.0%), Iguhu (88.0%), Bungoma (90.0%), Emakakha (93.4%), and Emutete (94.0%), whereas *An. arabiensis* was predominant in Kisian (64.4%) and Ahero (89.3%) (Table 1). The L1014F mutation was not detected in *An. gambiae* s.s or *An. arabiensis* mosquitoes at any sites. Frequency of L1014S point mutation was high for *An. gambiae* s.s. (85.8%–92.9%) except in the Kisian population (33.0%) (Table 2). For *An. arabiensis*, L1014S mutation frequency was lower (1.2%–39.1%). Homozygosity of L1014S genotype was high in *An. gambiae* s.s (30.0%–89.3%), but low in *An. arabiensis* (0–39.1%) (Table 2).

**Table 1:** *Anopheles* mosquitoes observed for insecticide resistance in 7 study sites, Western Kenya, 2012–2013

Study site	No. collected	% *An. arabiensis*	% *An. gambiae* subsp.	% Not amplified
Ahero	56	89.3	5.4	5.4
Kisian	225	64.4	32.9	2.4
Chulaimbo	100	24.0	72.0	4.0
Emutete	200	3.5	94.0	2.5
Emakakha	61	3.3	93.4	3.3
Iguhu	300	8.0	88.0	4.0
Bungoma	60	3.3	90.0	6.7

**Table 2:** Distribution of knockdown resistance genotypes and mutation frequencies by *Anopheles* mosquito species and study sites of pyrethroid- and DDT-resistant, organophosphate-susceptible *Anopheles* mosquitoes, Western Kenya, 2012–2013*

Study site	*An. gambiae*						*An. arabiensis*				
	No.	LL	LS	SS	Frequency, %		No.	LL	LS	SS	Frequency, %
Ahero	ND	ND	ND	ND	ND		50	46	4	0	4.0
Kisan	50	32	3	15	33.0		42	41	1	0	1.2
Chulaimbo	56	2	4	50	92.9		23	14	0	9	39.1
Emutete	87	7	4	76	89.7		ND	ND	ND	ND	ND
Emakakha	57	1	7	49	92.1		ND	ND	ND	ND	ND
Iguhu	108	10	7	91	87.5		16	15	1	0	3.1
Bungoma	53	5	5	43	85.8		ND	ND	ND	ND	ND

*LL, wild genotype at L1014 codon; SS, homozygous genotype for L1014S mutation; LS, heterozygous genotype; Frequency, allele frequency of L1014S mutation. ND, not done because of insufficient number of specimens.

We found through a survey that pyrethroids were the most frequently used insecticide for mosquito control (Technical Appendix Table). Pyrethroids were also frequently used for control of livestock disease vectors and agricultural pests. Most (73.3%–96.6%) surveyed households used pyrethroids for malaria vector control in the form of ITNs and IRS (Technical Appendix Table). Carbamate was mainly used for livestock disease vector control, and organophosphate was used seasonally for crop pest control among a small proportion of households.

## Conclusion

This study found high resistance to pyrethroid insecticide in all 7 study populations, no resistance to organophosphate, and patchy distribution of resistance to carbamate insecticide in *An. gambiae* and *An. arabiensis* mosquitoes in western Kenya. This finding has critical implications in guiding malaria vector control in Kenya. In Kenya, current policy on IRS use of insecticides is limited to pyrethroids and DDT (*13*). Considering widespread pyrethroid resistance, non–pyrethroid-based vector control tools may be preferable. There is a growing debate among government decision makers on whether to use organophosphates (such as malathion or chlorpyrifos methyl) and carbamates (such as bendiocarb) for IRS in Kenya. Our finding on the complete susceptibility to organophosphates in malaria vectors suggests that organophosphates are a potentially effective insecticide for IRS. The patchy distribution of resistance to carbamates calls for careful resistance baseline monitoring if carbamates are considered for IRS.

Although we detected widespread and strong phenotypic resistance to pyrethroids in *An. gambiae* mosquitoes, whether this resistance could result in operational ITN or IRS malaria control failure in the field is unknown. A report from Côte d’Ivoire showed that ITNs remained effective in reducing entomological inoculation rate in an area of higher *kdr* frequency in *An*. *gambiae* mosquitoes (*14*). Similarly, a cohort study in Malawi found that the use of ITNs reduced the incidence of cases of malaria by 30% in children in an area that has documented moderate levels of pyrethroid resistance and considerable malaria transmission (*15*). Cost-effectiveness is another consideration. A thorough assessment of the effect of resistance to pyrethroids on the efficacy and cost-effectiveness of LLINs and IRS for malarial disease and transmission will clarify the need to consider a shift from pyrethroids to alternative carbamate or organophosphate insecticides or to other integrated strategies to control malaria.

Technical Appendix Pesticide usage and knockdown rates by insecticides at individual study sites.
