# Supplementary material for: Pyrethroid and DDT Resistance and Organophosphate Susceptibility among Anopheles spp. Mosquitoes, Western Kenya
Source: Emerg Infect Dis. 2015 Dec;21(12):2178–81. doi: 10.3201/eid2112.150814 (PMC4672417; doi:10.3201/eid2112.150814)
Supplement: Technical Appendix — Pesticide usage and knockdown rates by insecticides at individual study sites. [file 15-0814-Techapp-s1.pdf]

Article DOI: http://dx.doi.org/10.3201/eid2112.150814

Pyrethroid and DDT Resistance and Organophosphate Susceptibility among *Anopheles* spp. Mosquitoes, Western Kenya

Technical Appendix

Technical Appendix Table. Pesticide/insecticide usage at different sites in Western Kenya

Site	Purpose	Insecticide class	N	Frequency
Bungoma	Livestock vector control	Carbamate	15/30	Monthly
		Pyrethroids	5/30	Monthly
	Crop pest control	Carbamate	10/30	Twice a year
		Organophosphate	4/30	Twice a year
	Mosquito control	Pyrethroids	23/30	Daily
Ahero	Livestock vector control	Carbamate	10/30	Bi-monthly
	Crop pest control	Pyrethroid	24/30	Twice a year
		Carbamate	20/30	Twice a year
		Organophosphate	5/30	Twice a year
	Mosquito control	Pyrethroids	27/30	Daily
Iguhu	Livestock vector control	Carbamate	20/30	Monthly
		Pyrethroid	19/30	Monthly
	Crop pest control	Organophosphate	17/30	Twice a year
	Mosquito control	Pyrethroids	29/30	Daily
Emutete	Livestock vector control	Carbamate	15/30	Monthly
		Pyrethroid	15/30	Monthly
	Mosquito control	Pyrethroids	22/30	Daily
Chulaimbo	Livestock vector control	Carbamate	10/30	Monthly
		Organophosphate	12/30	Bi-monthly
		Pyrethroid	9/30	Seasonally
	Mosquito control	Pyrethroids	24/30	Daily
Kisian	Livestock vector control	Carbamate	17/30	Monthly
		Pyrethroid	10/30	Seasonally
	Mosquito control	Pyrethroids	24/30	Daily

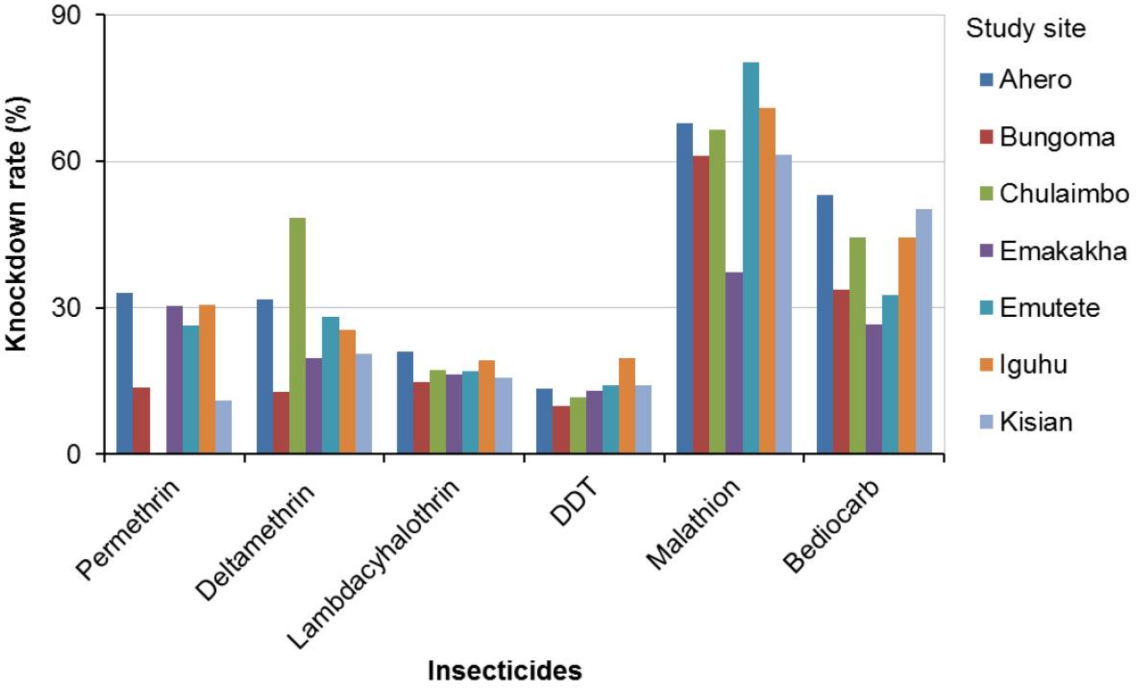

Technical Appendix Figure. Knockdown rates (observed after 60 min exposure) associated with different insecticides at different study sites in Western Kenya.
